# Effects of dependent care theory-based post-surgical home care intervention on self-care, symptoms, and caregiver burden in patients with primary brain tumor and their caregivers: a randomized controlled trial

**DOI:** 10.1007/s00520-024-08488-1

**Published:** 2024-04-18

**Authors:** Derya Dağdelen, Handan Zincir

**Affiliations:** Department of Public Health Nursing, Faculty of Health Sciences, Erciyes University, 38280 Kayseri, Turkey

**Keywords:** Caregiving burden, Home care, Nursing, Primary brain tumors, Self-care/dependent care, Self-care agency

## Abstract

**Purpose:**

This study aimed to examine the effect of dependent care theory-based post-surgical home care intervention on self-care, symptoms, and caregiver burden in primary brain tumor patients and their caregivers.

**Methods:**

A parallel-group randomized controlled trial was conducted with patients who underwent surgery for a primary brain tumor between March 2019 and January 2020 in a tertiary hospital and with caregivers who cared for them at home. Eligible patients and caregivers were determined by block randomization. Outcome measures included validated measures of self-care agency (Self-Care Agency Scale), symptoms and interference by symptoms (MD Anderson Symptom Inventory Brain Tumor-Turkish Form), and caregiver burden (Caregiver Burden Scale). Two-way analysis of variance was used in repeated measurements from general linear models compared to scale scores.

**Results:**

Self-care agency was significantly higher in the intervention group than in the control group in the first and sixth months after surgery (*p* < 0.05). The severity of the patients’ emotional, focal neurologic, and cognitive symptoms and interference by symptoms were significantly lower in the intervention group than in the control group (*p* < 0.05). Caregiver burden was significantly lower in the intervention group in the first, third, and sixth months after surgery (*p* < 0.05).

**Conclusion:**

Dependent care theory-based post-surgical home care intervention increased patients’ self-care and reduced symptoms and their effects. It also reduced the caregiver burden. Dependent care theory can guide the nursing practices of nurses who provide institutional and/or home care services to patients with chronic diseases and their caregivers.

**Trial Registration:**

NCT05328739 on April 14, 2022 (retrospectively registered).

**Supplementary Information:**

The online version contains supplementary material available at 10.1007/s00520-024-08488-1.

## Introduction

Brain and central nervous system (CNS) cancers are rare [1] but are responsible for significant morbidity and mortality worldwide and have increased in incidence [2]. The age-standardized incidence rate is 3.9 in males and 3.0 in females [3]. In Türkiye, the age-standardized incidence rate is 5.2 (per 100,000) for males and 4.2 (per 100,000) for females [4], which is above the world average.

Whether primary brain tumors (PBTs) are malignant or benign, patients experience many symptoms. Their effects continue after surgical treatment. Symptoms trigger each other, and more than one symptom disrupts individuals’ physical, cognitive, and psychosocial functions in a way that affects daily life [5–7]. Many challenges remain in the effective management of symptoms in adults with brain tumors [8]. Patients may become dependent on others before and after surgery [5]. When the caregiver burden studies in caregivers of patients with PBT were reviewed, it was found that the neuropsychological status of the patients [9], activities of daily living [10], and economic inadequacies [11] increase the caregiver burden and cause many problems in caregivers [12]. Studies conducted with PBT patients and their caregivers have suggested that effective interventions should be developed to meet their needs [13–15].

Orem’s Self-Care Deficit Nursing Theory (SCDNT) is one of the most frequently used theories in nursing practice [16]. Dependent Care Theory (DCT), one of the four central theories of SCDNT, provides the opportunity to evaluate patients and caregivers. The role of nurses in self-care/dependent care practices becomes crucial in the shortening of hospitalization times and the transfer of care of individuals from institutions to society [17]. Nurse-led intervention programs that evaluate PBT patients and their caregivers in their own homes after surgical treatment are limited [18–22]. It is emphasized that it is essential to provide appropriate interventions to patients and caregivers in meeting the needs of care-related individuals. Providing information appropriate to individuals’ experiences and needs in providing care and support can increase success [23, 24]. No studies based on DCT were found that evaluated patients with PBT and their caregivers. PBTs are a disease that can cause the emergence of many intense and unmet therapeutic self-care demands in the patient and caregivers. Patients and their caregivers must be taught how to provide and maintain appropriate care in their homes and the pathological problems and harmful effects that may arise during treatment and care [25]. This study aimed to examine the effect of dependent care theory-based post-surgical home care intervention on self-care, symptoms, and caregiver burden in primary brain tumor patients and their caregivers.

We hypothesized that the dependent care theory-based post-surgical home care intervention could improve self-care, decrease patients’ severity of symptoms and interference by symptoms, and caregiver burden for patients with PBT and their caregivers.

## Methods

### Study design

This study was a parallel-group randomized controlled trial (ClinicalTrial.gov; registration number: NCT05328739).

### Participants

Criteria for patients inclusion in the study were living within the region’s borders, being aged ≥ 18 years old, being diagnosed with PBT (glial or meningeal and grade I–III), having KPS ≥ 50 points, and being able to read and communicate. Patients’ exclusion criteria were diagnosed as having metastatic brain tumor, a pituitary adenoma, having undergone emergency surgery, having a biopsy, and being in grade IV. Caregiver inclusion criteria were age ≥ 18 years, providing primary care for patients, and being able to read and communicate. Criteria for terminating the research process for participants were wanting to leave the research process, meeting one of the criteria for exclusion from the sample after the surgery, spending the home care and follow-up process in another province, and/or being unable to reach the individual.

### Sample size

**Fig. 1:**
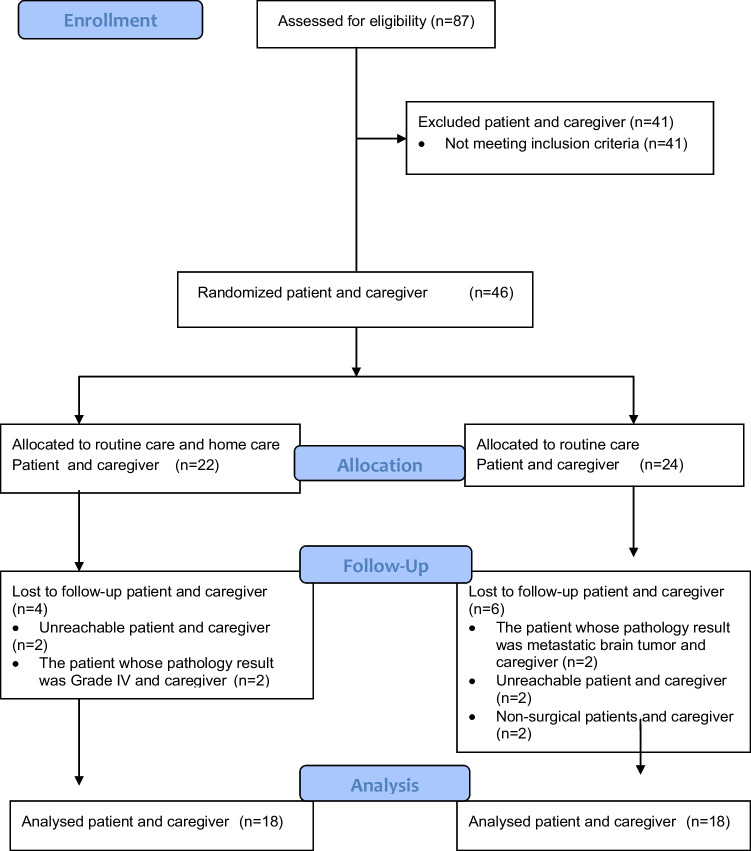
The study flowchart according to CONSORT 2010

G*Power 3.1.9.2 was used to calculate the sample size for this study. A similar study was used to determine the study’s sample [26]. The power of the study was 0.903 at the *α* = 0.05 level and 0.816 effect size. The study was conducted with 18 patients and 18 caregivers (Fig. 1).

### Randomization

Block randomization was used to balance the sample size between groups over time. A quadruple block structure consisting of six combinations was created. According to this structure created by an independent person from the research, intervention and control groups were generated by specifying which group the registered participant belonged to the researcher conducting the research process. Blinding was provided because patients and caregivers who met the inclusion criteria and agreed to participate in the study did not know which group they would join [27].

### Post-surgical home care intervention program

This program comprised the transition home after discharge and the 6-month post-surgical period for patients who underwent surgery for PBT and their caregivers. The program was constructed based on dependent care theory and had three purposes (Table 1). The first was to regulate, protect, and raise self-care agency; the second was to reduce the severity of symptoms and interference by symptoms; and the third was to reduce caregiver burden. In order to ensure the continuity of post-surgical care at home in 6 months, a supportive-developmental nursing system based on the DCT was used. This system, which includes education, counseling, and nursing care, focuses on goals. The content of the training booklet was created by determining the topics related to the postoperative self-care/dependent care demands and the practices that could meet them. The booklet for patients with PBT and their caregivers was prepared based on the literature on home care [17, 25, 26, 28–31]. The booklet, submitted for expert opinion, was found suitable by experts in the field (*W* = 0.267; *p* = 0.230). Nursing care was given to the patient and caregiver in line with the nursing diagnoses determined according to the self-care/dependent care demands of the patients and caregivers who had undergone craniotomy due to PBT [17, 25, 26, 28–30].

**Table 1:** Dependent care theory based on post-surgical home care program for patients with PBT and their caregivers

Aims: 1. Regulating, protecting, and raising self-care agency 2. Reducing the severity of symptoms and interference by symptoms 3. Reducing caregiver burden		
Nursing agency	Supportive-development nursing (counseling, education, nursing care)	Sources for patients and caregivers
Determine if patients and caregivers’ self-care/dependent care demands	✓ After surgery, maintaining adequate air, water, and food intake at home Nursing diagnoses: strengthening fluid balance, lack of self-care in oral hygiene, inadequate and/or unbalanced nutrition ✓ After surgery, ensuring adequate elimination and excretion at home Nursing diagnoses: functional urinary incontinence, risk of constipation, constipation ✓ After surgery, maintaining the balance between activity and rest at home Nursing diagnoses: risk of activity intolerance, activity intolerance, insufficient physical activity, pain, disruption in sleep pattern, fatigue, lack of self-care in bathing, lack of self-care in dressing, lack of self-care in meeting toilet needs, risk of ineffective cerebral tissue perfusion, risk of deterioration in home care ✓ After surgery, prevention of hazards at home Nursing diagnoses: risk of infection, delay in surgical recovery, fall/injury risk, risk of seizure, bleeding risk ✓ After surgery, maintaining the balance between loneliness and social interaction at home Nursing diagnoses: fear, anxiety, deterioration in mood, social isolation, disruption in social interaction, distortion in body image, decreased self-esteem, ineffective role performance, change in family process, spiritual distress, disruption in family coping, risk of strain in the caregiver role, strain in the caregiver role	Training booklet Home visits Face-to-face interaction Telephone contacts
Support to caregiver	✓ Ability to decide where care will be provided for the patient ✓ Ability to make safe and healthy environmental arrangements for the patient ✓ Ability to determine and meet care needs ✓ Ability to develop, change, and balance self-care agency ✓ Ability to balance self-care/ dependent care roles ✓ Ability to collaborate and communicate	Training booklet Home visits Face-to-face interaction Telephone contacts
Develop and protect power components for self-care/dependent care	✓ Self-confidence and respect ✓ Controlling physical energy ✓ Motivation ✓ Ability to make decisions about self-care ✓ Ability to acquire and apply knowledge ✓ Cognitive status and communication skills ✓ Ability to integrate self-care behaviors into individual and social life	Training booklet Home visits Face-to-face interaction Telephone contacts

### Procedures

Patients and caregivers were recruited at the same time between March 2019 and January 2020 in a tertiary hospital in Türkiye. After the decision for surgery, the patient and caregiver were interviewed and informed about the study, the Informed Voluntary Consent Form-IVCF was filled out, and initial data were collected (preoperative period-T0). Block randomization assigned patients and caregivers to intervention and control groups. Patients and caregivers in the control group received routine care in the hospital until discharge. Three home visits were made to collect the data of patients and caregivers in the first (postoperative period-T1), third (postoperative period-T2), and sixth (postoperative period-T3) months of surgery after discharge, but no intervention was made.

Patients and caregivers in intervention groups received training and a training booklet until patients were discharged. This total time lasted 90–120 min. In order to prepare the patient and caregiver for the transition home, training was provided on the self-care/dependent care demands that may be encountered in the first week at home and how they can be met, where care can be given and necessary environmental arrangements, what to do in emergencies and drug treatment. Two home visits were made within the first month, planned for education, counseling, and nursing care. The first home visit was made the week after discharge (10–18 days after surgery). At this stage, the patient and caregiver were evaluated in their environment, and nursing care was given for the needs and problems determined for the first visit. At the second home visit (30–40 days after surgery), planned and made at the end of the first month, the patient and caregiver were evaluated in their environment, and nursing care was given for the needs and problems determined for the second visit. Then, in the home visits made once in the second, third, and fourth months, the patient and caregiver were evaluated in their environment similarly, and nursing care was given for the needs and problems determined for that visit. No home visits were made between the fourth and sixth months, but telephone counseling was provided when necessary. The last home visit was made for the sixth-month measurements and goodbye to the patient and caregivers. In this process, nursing care was given to the patient and caregiver per the nursing diagnoses determined according to patients’ and caregivers’ self-care/dependent care demands.

### Measurements

Self-care was measured using the Self-Care Agency Scale (SCAS) [32], and the severity of symptoms in patients with PBT and the life-threatening condition of patients were measured using the MD Anderson Symptom Inventory Brain Tumor-Turkish Form (MDA-BTSETr) [33]. Caregiver burden was measured using the Caregiver Burden Scale (CBS) [34]. These measurement tools are valid and reliable tools suitable for Turkish society. Cronbach’s alpha value of the measurement tools in this study was acceptable, at 0.94 in patients and 0.91 in caregivers for SCAS; 0.85 and 0.88 for MDA-BTSE^Tr^ and CBS, respectively. The Karnofsky Performance Scale (KPS) [35] allows the evaluation of the patients’ individual and medical care needs to obtain information about symptom severity and level of function at work and home.

### Statistical analysis

IBM SPSS 26 was used for statistical analyses. Descriptive statistics are given as number of units (*n*), percentage (%), and mean ± standard deviation. The normal distribution of the data of numerical variables was evaluated with the Shapiro–Wilk test of normality and Q-Q graphs. Homogeneity of variances was evaluated with the Levene test, and two-way analysis of variance was used in repeated measurements from general linear models in comparison of scale scores between T0, T1, T2, and T3 between groups and within groups. Bonferroni correction was applied when comparing the main effects. Comparisons between categorical variables and groups were evaluated with Fisher’s exact test in 2 × 2 and r × c tables [36], *p* < 0.05 value was considered statistically significant.

## Results

### Participant characteristics

Comparisons of patients’ and caregivers’ descriptive and clinical characteristics are shown in Table 2; there were no differences between the two groups (*p* > 0.05), indicating that they were comparable.

**Table 2:** Comparison of the patients’ and caregivers ‘descriptive and clinical characteristics

Variable	Intervention group (*n* = 9), *n* (%)	Control group (*n* = 9), *n* (%)	*p*^†^	Variable	Intervention group (*n* = 9), *n* (%)	Control group (*n* = 9),* n* (%)	*p*^†^
Patients				Caregivers			
Gender			1.000	Gender			1.000
Female	6 (66.7)	5 (55.6)		Female	7 (77.8)	6 (66.7)	
Male	3 (33.3)	4 (44.4)		Male	2 (22.2)	3 (33.3)	
Marital status			1.000	Marital status			0.471
Married	8 (88.9)	9 (100.0)		Married	7 (77.8)	9 (100.0)	
Single	1 (11.1)	0 (0.0)		Single	2 (22.2)	0 (0.0)	
Education status				Education status			0.418
Literate	1 (11.1)	1 (11.1)		Literate	0 (0.0)	1 (11.2)	
Primary school graduate	2 (22.2)	6 (66.7)	0.333	Primary school graduate	4 (44.4)	4 (44.4)	
Secondary school graduate	2 (22.2)	1 (11.1)		Secondary school graduate	1 (11.2)	0 (0.0)	
High school graduate	3 (33.3)	0 (0.0)		High school graduate	0 (0.0)	2 (22.2)	
Undergraduate	1 (11.1)	1 (11.1)		Undergraduate	4 (44.4)	2 (22.2)	
Working status			1.000	Working status			1.000
Working	3 (33.3)	3 (33.3)		Working	3 (33.3)	2 (22.2)	
Not working	6 (66.7)	6 (66.7)		Not working	6 (66.7)	7 (77.8)	
Type of tumor			1.000	Chronic disease status			0.576
Glioma	3 (33.3)	2 (22.2)		Yes	1 (11.1)	3 (33.3)	
Meningioma	6 (66.7)	7 (77.8)		No	8 (88.9)	6 (66.7)	
Tumor grade				Place of residence			1.000
Grade-1	6 (66.7)	7 (77.8)	0.576	City center	7 (77.8)	6 (66.7)	
Grade-2	1 (11.1)	2 (22.2)		District	2 (22.2)	3 (33.3)	
Grade-3	2 (22.2)	0 (0.0)					
Tumor resection			1.000	Role in family			1.000
Total	7(77.8)	8 (88.9)		Child	3 (33.3)	4 (44.4)	
Subtotal	2 (22.2)	1 (11.1)		Sibling Spouse	1 (11.1) 5 (55.6)	0 (0.0) 5 (55.6)	
Treatment			0.471	Support from relatives,			1.000
S	7 (77.8)	9 (100.0)		friends, and neighbors			
S + RT	1 (11.1)	0 (0.0)		Yes	8 (88.9)	9 (100.0)	
S + RT + CT	1 (11.1)	0 (0.0)		No	1 (11.1)	0 (0.0)	
T0-KPS							
50–70	4 (44.4)	3 (33.3)	0.367				
80–100	5(55.6)	6 (66.7)					
T1–KPS							
50–70	0 (0.0)	3 (33.3)	0.367				
80–100	9 (100.0)	6 (66.7)					
T2-KPS							
50–70	0 (0.0)	1 (11.1)	0.367				
80–100	9 (100.0)	8 (88.9)					
T3-KPS			-				
50–70	0 (0.0)	0 (0.0)					
80–100	9 (100.0)	9 (100.0)					

^†^Fisher’s exact test, *S* surgical, *RT* radiotherapy, *CT* chemotherapy, *KPS* Karnofsky Performance Scale, *T0* preoperative period, *T1* postoperative period-1st month, *T2* postoperative period-3rd month, *T3* postoperative period-6th month

### Intervention effects for primary brain tumor patients

Comparison of the mean scores of SCAS of the patients in the control and intervention groups according to the measurement times are shown in Table 3. At baseline, SCAS scores for patients in the intervention and control groups were similar (*p* > 0.05). General linear model analysis showed a significant effect of group on SCAS score (*p* = 0.45) and the effect of time (*p* = 0.006). SCAS scores for patients in the intervention group were higher than in the control group in the first and sixth months after the surgery (*p* < 0.05). These results suggest that patients in the intervention group had increased SCAS scores after the intervention.

**Table 3:** Comparison of self-care agency mean scores of patients in the intervention and control groups by measurement times

Measurement times	Intervention groups, mean (SD)	Control groups, mean (SD)	Between groups, TS			Model statistics^§^		
			*F*	*p*		Effect	*F*	*p*
Self-care agency								
T0^§§^	90.11 (21.97)^a, b^	84.22 (13.49)	0.469		0.503	Group	4.905	0.045
T1^§§^	101.11 (11.45)^*a, b*^	86.44 (15.21)	5.342		0.034	Time	4.738	0.006
T2^§§^	98.55 (8.02)^*a*^	88.22 (14.32)	3.566		0.077	Group x time	1.572	0.208
T3^§§^	104.44 (11.12)^*b*^	88.55 (13.96)	7.127		0.017			
*Within groups TS*	*F* = 4.173; *p* = 0.026	*F* = 0.391; *p* = 0.762						

^**§**^General linear models, two-way analysis of variance

^§ §^*a*, *b*: within groups Bonferroni’s correction multiple comparison test

*TS* test statistics, *T0* preoperative period, *T1* postoperative period-1st month, *T2* postoperative period-3rd month, *T3* postoperative period-6th month

Comparisons of the mean scores of the severity of symptoms and interference by symptoms of the patients in the control and intervention groups according to the measurement times are shown in Table 4. General linear model analysis showed a significant effect of time (*p* < 0.001) on emotional and focal neurologic symptom scores. Significant effects of group (*p* = 0.047), the effect of time (*p* < 0.001), and group x time (*p* = 0.008) on cognitive symptoms were also significant. Significant effects of time (*p* < 0.001) and group x time (*p* = 0.043) on interference by symptoms scores were also significant. These results suggest that patients in the intervention group had decreased severity symptoms and interference by symptoms scores after the intervention.

**Table 4:** Comparison of MDA-BTSE^Tr^ symptom mean scores of patients in the intervention and control groups by measurement times

Measurement times	Intervention groups, mean (SD)	Control groups, mean (SD)	Between groups, TS		Model statistics^†^		
			*F*	*p*	Effect	*F*	*p*
Emotional							
T0^††^	4.49 (1.11)^*a*^	2.95(2.20)^*a*^	3.475	0.081	Group	0.406	0.533
T1^††^	3.40 (2.13)^*a*^	3.40(2.00)^*a, c*^	0.000	1.000	Time	16.397	< 0.001
T2^††^	1.64 (1.90)^*b*^	1.89(1.55)^*a*^	0.090	0.768	Group x time	1.329	0.277
T3^††^	0.67 (1.04)^*b*^	0.67 ± 0.98^*b*^	0.000	1.000			
*Within groups TS*	*F* = 23,384; *p* < 0.001	*F* = 15,623; *p* < 0.001					
Cognitive							
T0^††^	2.90 (1.54)^*a*^	1.11 (1.32)	7.030	0.017	Group	4.633	0.047
T1^††^	0.47 (1.09)^*b*^	0.33 (0.83)	0.092	0.765	Time	20.692	< 0.001
T2^††^	0.19 (0.58)^*b*^	0.11 (0.33)	0.138	0.715	Group x time	4.478	0.008
T3^††^	0.00 (0.00)^*b*^	0.00 (0.00)	-	-			
*Within groups TS*	*F* = 12.298; *p* < 0.001	*F* = 2.446; *p* = 0.107					
Focal neurologic							
T0^††^	2.53 (2.42)^*a*^	2.14 (2.36)^*a, b*^	0.119	0.735	Group	0.329	0.574
T1^††^	1.05 (0.97)^*a, b*^	1.80 (1.21)^*a*^	2.096	0.167	Time	11.196	< 0.001
T2^††^	0.30 (0.37)^*a, b*^	0.69 (0.55)^*b*^	3.051	0.100	Group x time	0.632	0.598
T3^††^	0.00 (0.00)^*b*^	0.03 (0.08)^*c*^	1.000	0.332			
*Within groups TS*	*F* = 5.044; *p* = 0.014	*F* = 9.798; *p* < 0.001					
Treatment evaluation							
T0^††^	2.26 (2.14)^*a*^	0.63 (0.85)	4.514	0.049	Group	1.490	0.240
T1^††^	1.18 (1.97)^*a, b*^	0.89 (1.59)	0.122	0.731	Time	2.301	0.089
T2^††^	1.04 (1.41)^*a, b*^	0.74 (0.72)	0.318	0.581	Group x time	1.864	0.148
T3^††^	0.33 (0.58)^*b*^	0.48 (0.73)	0.224	0.643			
*Within groups TS*	*F* = 4.352; *p* = 0.023	*F* = 0.469; *p* = 0.709					
General							
T0	1.05 (0.90)	1.00 (1.16)	0.013	0.911	Group	0.013	0.911
T1	0.55 (0.57)	0.55 (0.69)	0.000	1.000	Time	2.721	0.118
T2	0.30 (0.39)	0.39 (0.50)	0.155	0.699	Group x time	0.046	0.987
T3	0.05 (0.17)	0.11 (0.22)	0.364	0.555			
*Within groups TS*	*F* = 2.928; *p* = 0.115	*F* = 2.470; *p* = 0.121					
Gastrointestinal							
T0	2.50 (3.95)	0.55 (1.10)	2.021	0.174	Group	1.820	0.196
T1	0.22 (0.67)	0.22 (0.67)	0.000	1.000	Time	4.462	0.051
T2	0.00 (0.00)	0.00 (0.00)	-	-	Group x time	1.955	0.133
T3	0.00 (0.00)	0.00 (0.00)	-	-			
*Within groups TS*	*F* = 3.349; *p* = 0.063	*F* = 0.565; *p* = 0.580					
Interference by symptoms							
T0^††^	4.78 (1.64)^*a*^	3.74 (2.05)^*a, b*^	1.401	0.254	Group	0.273	0.609
T1^††^	4.28 (1.05)^*a*^	4.59 (1.07)^*a*^	0.398	0.537	Time	27.274	< 0.001
T2^††^	2.24 (1.63)^*b*^	3.13 (0.87)^*b*^	2.090	0.168	Group x time	2.924	0.043
T3^††^	1.18 (1.00)^*c*^	1.98 (1.17)^*c*^	2.404	0.141			
*Within groups TS*	*F* = 58.472; *p* < 0.001	*F* = 31.620; *p* < 0.001					

^†^General linear models, two-way analysis of variance test

^††^*a*, *b*, c: within groups Bonferroni’s correction multiple comparison test

*TS* test statistics, *T0* preoperative period, *T1* postoperative period-1st month, *T2* postoperative period-3rd month, *T3* postoperative period-6th month

### Intervention effects for caregivers

Comparisons of the mean scores of SCAS and CBS according to the measurement times of the caregivers in the control and intervention groups are shown in Table 5. General linear model analysis showed no significant effects of the group, the effect of time, and group x time (*p* > 0.05) on the SCAS score. These results suggest that the intervention does not affect caregivers’ SCAS scores. Significant effects of group (*p* = 0.005), the effect of time (*p* < 0.001), and group x time (*p* < 0.001) on CBS scores were also significant. According to model statistics, in the change of the mean scores of CBS over time, the decrease in the intervention group’s scores was higher than that of the control group (*p* < 0.001).

**Table 5:** Comparison of self-care agency and caregiver burden mean scores of caregivers in the intervention and control groups by measurement times

Measurement times	Intervention groups, mean (SD)	Control groups, mean (SD)	Between groups, TS		Model statistics^†^		
			*F*	*p*	Effect	*F*	*p*
Self-care agency							
T0	103.44 (11.48)	92.33 (18.08)	2.423	0.139	Group	1.254	0.279
T1	102.22 (10.07)	95.33 (24.12)	0.625	0.441	Time	0.449	0.719
T2	102.11 (10.47)	97.89 (17.44)	0.388	0.542	Group x time	0.360	0.782
T3	105.00 (10.04)	98.11 (21.72)	0.746	0.400			
*Within groups TS*	*F* = 0.603; *p* = 0.624	*F* = 0.465; *p* = 0.711					
Caregiver burden							
T0^††^	23.44 (10.14)^*a*^	26.55 (11.38)^*a, c*^	0.375	0.549	Group	10.849	0.005
T1^††^	24.55 (9.96)^*a*^	46.78 (5.45)^*b*^	34.460	< 0.001	Time	22.186	< 0.001
T2 ^††^	18.33 (11.57)^*a*^	35.44 (11.08)^*a*^	10.262	0.006	Group x time	6.798	0.001
T3^††^	12.11 (11.55)^*b*^	23.00 (10.40)^*c*^	4.417	0.048			
*Within groups TS*	*F* = 12.042; *p* < 0.001	*F* = 58.510; *p* < 0.001					

^†^General linear models, two-way analysis of variance test

^††^*a*, *b*: within groups Bonferroni’s correction multiple comparison test

*TS* test statistics, *T0* preoperative period, *T1* postoperative period-1st month, *T2* postoperative period-3rd month, *T3* postoperative period-6th month

## Discussion

In this study, we examined the effect of a post-surgical home care intervention based on dependent care theory on patients with primary brain tumors and their caregivers and found that self-care increased and the severity of symptoms and interference by symptoms and caregiver burden decreased.

Our results showing increased patients’ self-care ability are similar to previous studies [20, 37–39]. The difference between this study and others is that the home care intervention, based on the dependent care theory, simultaneously supports the patient and the caregiver. In addition, it is thought that evaluating individuals in their environment through home visits, determining their care needs, and systematically planning and implementing education, counseling, and nursing care positively affect self-care skills.

Many patients with primary brain tumors experience symptoms before and after treatment that interfere with daily activities, mood, and tasks, including household chores, relationships with other people, walking, and enjoyment of life [6, 9, 33, 40–43]. It is essential because the symptoms are important severity and interference in individuals’ lives target therapeutic self-care needs and affect self-care/dependent care ability and nursing practices. After the intervention, the severity of emotional, focal neurological, and cognitive symptoms and interference by symptoms decreased in patients, similar to the previous report [26]. These results suggest that the resources created for patients and caregivers within the scope of the home care program, which is the road map in the study, are adequate.

After our intervention, there was no significant difference in caregivers’ self-care agency. This result is consistent with the results of Deek et al. [44]. This may be due to sample differences and intervention differences. Our results showing a decrease in the CBS of caregivers were similar to previous studies [45, 46], but there were also study results that were not similar [19, 22]. The difference between this study and others is that the home care intervention supports the patient and caregiver simultaneously. In the home care intervention, it was prioritized to develop the skills and abilities of caregivers, such as determining and meeting the needs of the patient, determining care priorities, applications for emergencies, evaluating the home environment and making the necessary arrangements, using support resources, and protecting and developing their own self-care agency. The significant decrease in the postoperative CBS scores of the caregivers in the intervention group compared to the control group showed that home care programs targeting both the patient and the caregivers contributed positively.

## Limitations

This study has some limitations. In the region where the research was conducted, brain surgeries were performed in a single center. Since both the patient and their caregivers were evaluated in the study, individuals who met the conditions of the research criteria at the same time were included in the study. Therefore, the research was conducted with a small group.

## Conclusion

Dependent care theory-based post-surgical home care intervention increased patients’ self-care and reduced symptoms and their effects. It also reduced the caregiver burden. Dependent care theory can guide the nursing practices of nurses who provide institutional and/or home care services to patients with chronic diseases and their caregivers.

### Supplementary information

Below is the link to the electronic supplementary material.

Supplementary file1 (DOC 217 KB)

## Data Availability

No datasets were generated or analysed during the current study.

## References

[1] Siegel RI, Miller KD, Wagle NK (2023). Cancer statistics, 2023. CA Cancer J Clin.

[2] GBD (2016). Brain and Other CNS Cancer Collaborators (2019) Global, regional, and national burden of brain and other CNS cancer, 1990–2016: a systematic analysis for the Global Burden of Disease Study 2016. Lancet Neurol.

[3] Sung H, Ferlay J, Siegel RL (2021). Global Cancer Statistics 2020: GLOBOCAN estimates of incidence and mortality worldwide for 36 cancers in 185 countries. CA Cancer J Clin.

[4] Türkyılmaz M, Oruç Hamavioğlu Eİ, Dündar S (2022) Türkiye cancer statistics 2018. Tolunay T, Kaygusuz S, Keskinkılıç B (eds) Republic of Türkiye Ministry of Health General Directorate of Public Health p.30

[5] Krajewski S, Furtak J, Zawadka-Kunikowska M (2023). Functional state and rehabilitation of patients after primary brain tumor surgery for malignant and nonmalignant tumors: a prospective observational study. Curr Oncol.

[6] Nassiri F, Suppiah S, Wang JZ (2020). How to live with a meningioma: experiences, symptoms, and challenges reported by patients. Neurooncol Adv.

[7] Tankumpuan T, Utriyaprasit K, Chayaput P (2015). Itthimathin P (2015) Predictors of physical functioning in postoperative brain tumor patients. J Neurosci Nurs.

[8] Zhang R, Wang DM, Liu YL (2023). Symptom management in adult brain tumours: a literature review. Nurs Open.

[9] Kanter C, D’Agostino NM, Daniels M (2014). Together and apart: providing psychosocial support for patients and families living with brain tumors. Support Care Cancer.

[10] Sherwood PR, Given BA, Given CW (2006). Predictors of distress in caregivers of persons with a primary malignant brain tumor. Res Nurs Health.

[11] Bayen E, Laigle-Donadey F, Proute M (2017). The multidimensional burden of informal caregivers in primary malignant brain tumor. Support Care Cancer.

[12] Sharma A, Kaur S, Tewari MK, Singh A (2014). Extent of the burden of caregiving on family members of neurosurgical inpatients in a tertiary care hospital in north India. J Neurosci Nurs.

[13] Heckel M, Hoser B, Stiel S (2018). Caring for patients with brain tumors compared to patients with non-brain tumors: experiences and needs of informal caregivers in home care settings. J Psychosoc Oncol.

[14] Piil K, Jakobsen J, Christensen KB (2018). Needs and preferences among patients with high-grade glioma and their caregivers - a longitudinal mixed methods study. Eur J Cancer Care (Engl).

[15] Scaratti C, Leonardi M, Saladino A (2017). Needs of neuro-oncological patients and their caregivers during the hospitalization and after discharge: results from a longitudinal study. Support Care Cancer.

[16] Meleis AI (2012) Theoritical nursing development & progress. Wolters Kluwer Health, Lippincott Williams & Wilkins

[17] Taylor SG, Renpenning KM (2011) Self-care science, nursing theory and evidence-based practice. Springer Publishing Company

[18] Boele FW, Rooney AG, Bulbeck H, Sherwood P (2019). Interventions to help support caregivers of people with a brain or spinal cord tumour. Cochrane Database Syst Rev.

[19] Dionne-Odom JN, Azuero A, Lyons KD (2015). Benefits of early versus delayed palliative care to ınformal family caregivers of patients with advanced cancer: outcomes from the ENABLE III Randomized Controlled Trial. J Clin Oncol.

[20] Liu C, Zhang X, Liu X (2020). Application of Orem’s self-care theory in the nursing of patients after craniocerebral tumor surgery and its impacts on their self-care ability and mental state. Int J Clin Exp Med.

[21] Pace A, Villani V, Di Pasquale A (2014). Home care for brain tumor patients. Neurooncol Pract.

[22] Reblin M, Ketcher D, Forsyth P, Mendivil E, Kane L, Pok J, Meyer M, Wu YP, Agutter J (2018). Outcomes of an electronic social network intervention with neuro-oncology patient family caregivers. J Neurooncol.

[23] Petruzzi A, Finocchiaro CY, Lamperti E, Salmaggi A (2013). Living with a brain tumor: reaction profiles in patients and their caregivers. Support Care Cancer.

[24] Sherwood PR, Cwiklik M, Donovan HS (2016). Neuro-oncology family caregiving: review and directions for future research. CNS Oncol.

[25] Repenning K, Taylor SG, Pickens JM (2016) Foundation of professional nursing: care of self and others. Springer Publishing Company, LLC

[26] Baksi Şimşek A (2013) Examination of adaptive and non-adaptive behaviours of the patients with primary brain tumor by Roy Adaptation Model, the effect of education on symptom and coping. Dissertation, Dokuz Eylul University

[27] Nahcivan N (2014) Quantitative research designs. In: Erdoğan S, Nahcivan N, Esin MN (Eds) Research in nursing: process, practice and critical. Nobel Medical Publishing. p.87–130

[28] American Brain Tumor Association (2018) About brain tumors, a primer for patient and caregivers. Retrieved from https://www.abta.org/wp-content/uploads/2020/06/About-Brain-Tumors_2020_web_en.pdf

[29] Karadakovan A, Özbayır T (2014) Brain tumors In: Karardakovan A, Eti Aslan F (Eds) Care in internal and surgical diseases .Academic Medical Publishing. p. 1184–1197.

[30] Wilkson J, Barcus L (2018) Pearson handbook of nursing diagnoses. Kapucu S, Akyar I, Korkmaz F (Translation editors), (11th edition) Pelikan Publishing.

[31] Kaya H, Trans (2014). Care in patients with intracranial tumors. Topçuoğlu MA, Durna Z, Karadakovan A.

[32] Nahcivan NO (2004). A Turkish language equivalence of the exercise of self-care agency scale. West J Nurs Res.

[33] Baksi A, Dicle A (2010). MD The validity and reliability of MD Anderson Symptom Inventory-Brain Tumor. Dokuz Eylül Univer Nurs Faculty Electronic J.

[34] İnci FH, Erdem M (2008). Validity and reliability of the burden interview and its adaptation to Turkish. J Anatolia Nurs Health Sci.

[35] Schaafsma J, Osoba D (1994). The Karnofsky performance status scale re-examined: a cross-validation with the EORTC-C30. Qual Life Res.

[36] Mehta CR, Patel NR (1986). A hybrid algorithm for Fisher’s exact test in unordered rxc contingency tables. Commun Statistics-Theory Methods.

[37] Altın Çetin A (2020) The effect of telephone symptom triage protocol on symptom management, quality of life and self care agency in patients with cancer who applied systemic treatment. Dissertation, Akdeniz University

[38] Ceylan, H. (2020). The effect of web-based education based on Self-Care Deficiency Theory on self-care power, self-efficacy and perceived social support in patients undergoing peritoneal dialysis. Dissertation, Akdeniz University

[39] Mohammadpour A, Sharghi NR, Khosravan S (2015). The effect of a supportive educational intervention developed based on the Orem’s self-care theory on the self-care ability of patients with myocardial infarction: a randomised controlled trial. J Clin Nurs.

[40] Cahill JE, Lin L, LoBiondo-Wood G (2014). Personal health records, symptoms, uncertainty, and mood in brain tumor patients. Neurooncol Pract.

[41] Dhandapani M, Gupta S, Mohanty M (2017). Prevalence and trends in the neuropsychological burden of patients having ıntracranial tumors with respect to neurosurgical intervention. Ann Neurosci.

[42] Schepers VPM, van der Vossen S, van der Berkelbach Sprenkel JW (2018). Participation restrictions in patients after surgery for cerebral meningioma. J Rehabil Med.

[43] Armstrong TS, Mendoza T, Gning I (2006). Validation of the MD Anderson symptom inventory brain tumor module (MDASI-BT). J Neurooncol.

[44] Deek H, Chang S, Newton PJ (2017). An evaluation of involving family caregivers in the self-care of heart failure patients on hospital readmission: randomised controlled trial (the FAMILY study). Int J Nurs Stud.

[45] Bayrak B (2019) The effect of training provided to home caregivers of patients who received hemodialysis treatment upon care burden and patients’ quality of life. Dissertation, Karadeniz Technical University

[46] Bitek DE (2019) The effect of post-stroke discharge training and telephone counseling on the functional status of patients and the burden of care for their relatives. Dissertation, Trakya University

